# Antimicrobial Polymers: The Potential Replacement of Existing Antibiotics?

**DOI:** 10.3390/ijms20112747

**Published:** 2019-06-04

**Authors:** Nor Fadhilah Kamaruzzaman, Li Peng Tan, Ruhil Hayati Hamdan, Siew Shean Choong, Weng Kin Wong, Amanda Jane Gibson, Alexandru Chivu, Maria de Fatima Pina

**Affiliations:** 1 Faculty of Veterinary Medicine, Locked bag 36, Universiti Malaysia Kelantan, Pengkalan Chepa 16100, Kelantan, Malaysia; li.peng@umk.edu.my (L.P.T.); ruhil@umk.ed.my (R.H.H.); shean.cs@umk.edu.my (S.S.C.); 2 School of Health Sciences, Universiti Sains Malaysia, Kubang Kerian 16150, Kelantan, Malaysia; wengkinwong@usm.my; 3 Royal Veterinary College, Pathobiology and Population Sciences, Hawkshead Lane, North Mymms, Hatfield AL9 7TA, UK; ajgibson@rvc.ac.uk; 4 UCL Centre for Nanotechnology and Regenerative Medicine, Division of Surgery & Interventional Science, University College London, London NW3 2PF, UK; a.chivu.14@ucl.ac.uk; 5 Medicines and Healthcare Regulatory Products Agency, 10 South Colonnade, Canary Wharf, London E14 4PU, UK; mfatimagpina@gmail.com

**Keywords:** antimicrobial resistance, antimicrobial polymers, ESKAPE pathogens

## Abstract

Antimicrobial resistance is now considered a major global challenge; compromising medical advancements and our ability to treat infectious disease. Increased antimicrobial resistance has resulted in increased morbidity and mortality due to infectious diseases worldwide. The lack of discovery of novel compounds from natural products or new classes of antimicrobials, encouraged us to recycle discontinued antimicrobials that were previously removed from routine use due to their toxicity, e.g., colistin. Since the discovery of new classes of compounds is extremely expensive and has very little success, one strategy to overcome this issue could be the application of synthetic compounds that possess antimicrobial activities. Polymers with innate antimicrobial properties or that have the ability to be conjugated with other antimicrobial compounds create the possibility for replacement of antimicrobials either for the direct application as medicine or implanted on medical devices to control infection. Here, we provide the latest update on research related to antimicrobial polymers in the context of ESKAPE (*Enterococcus faecium*, *Staphylococcus aureus*, *Klebsiella pneumoniae*, *Acinetobacter baumannii*, *Pseudomonas aeruginosa*, and *Enterobacter* spp.) pathogens. We summarise polymer subgroups: compounds containing natural peptides, halogens, phosphor and sulfo derivatives and phenol and benzoic derivatives, organometalic polymers, metal nanoparticles incorporated into polymeric carriers, dendrimers and polymer-based guanidine. We intend to enhance understanding in the field and promote further work on the development of polymer based antimicrobial compounds.

## 1. Introduction

**Figure 1:**
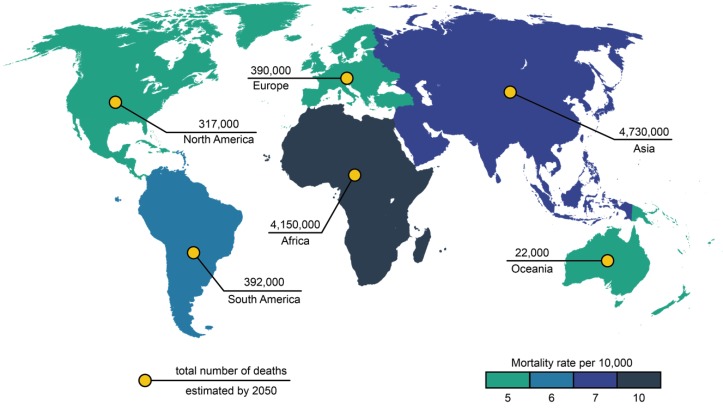
The estimated number of deaths at every continent in 2050 attributed to antimicrobial resistance (AMR). Image adapted from [2].

Antimicrobial resistance (AMR) is currently widespread across 22 countries with an estimated 500,000 people infected worldwide [1]. A report by O’Neill and colleagues has estimated 10 million deaths in 2050 will be due to AMR (Figure 1) [2]. Such data informed and shaped the Global Action Plan on Antimicrobial Resistance and has encouraged governments and public health agencies to increase efforts in AMR surveillance and research. To date, 52 countries (25 high-income, 20 middle-income and 7 low-income countries) have provided information about their national surveillance systems and data on levels of AMR [2].

This surveillance and data are important in monitoring and clarifying the epidemiology of AMR, in order to allow priorities to be set and to develop public health policy and strategies targeting this global concern. In this first section of the review, we provide the current epidemiology of multidrug-resistant organisms (MDROs) globally, focusing mainly on ESKAPE pathogens (*Enterococcus faecium*, *Staphylococcus aureus*, *Klebsiella pneumoniae*, *Acinetobacter baumannii*, *Pseudomonas aeruginosa*, and *Enterobacter* spp.). These ESKAPE pathogens are capable of “escaping” from common antibacterial treatments and have been listed as World Health Organization (WHO) priority pathogens with critical and high priority [3]. Although extensive studies on the prevalence of antimicrobial resistance pathogens were conducted, these studies were largely limited to certain countries and we believe that these data are not able to showcase the overall picture of antimicrobial resistance around the globe. Hence, we sought to evaluate the current global prevalence of ESKAPE pathogens by reviewing published work performed between the years 2013–2018 (Table 1).

The claim on the global prevalence status of AMR among ESKAPE pathogens is rather challenging as standardisation of resistance testing across antibiotic groups being employed by the available studies is lacking. In order to give a simple visualization on the current global prevalence of antimicrobial resistance among ESKAPE pathogens, we compiled a meta-analysis of works conducted in different geographical locations and classified them into 4 major classes namely, susceptible, resistant; highly resistant and extremely resistant by having 50% of the organism show resistance against none, one or more of the 13 antibiotic groups stated.

According to the summarized work in Table 1, *A. baumannii* is reported as extremely resistant i.e., 50% resistant to at least 3 drug classes for all selected countries. It is considered one of the most challenging ESKAPE pathogens due to its particular antimicrobial resistance characteristics, having developed resistance to almost all known antimicrobials [14]. The increasing trend of multidrug resistant (MDR) pathogens has diminished the options of effective therapeutic drugs for bacterial infection. The return to the previously abandoned antimicrobial, colistin, considered to be the ‘last resort’ treatment is also challenging as emerging resistant clinical isolates to colistin has been reported globally [15,16].

Limiting overuse and misuse of antimicrobials were proposed as the solution to limit and even reduce the MDR pathogens. However, the theory of reduction in resistance in the absence of a given antimicrobial is no longer a novel approach [17]. Although theoretically attractive, the reversibility of resistance has proven difficult in practice as the success rate is highly dependent on many other factors such as the fitness cost of the resistance mechanism, the epidemic potential of the bacteria/strain, and the transmission route of the species [18]. Efforts to develop new antimicrobials concurrently have diminished due to a combination of market forces and the inability to match the fast-paced growth of antimicrobial resistance in superbugs [19].

Decisively, although antimicrobial resistance does not seem obviously reversible, efforts must still be focused on imposing measures that might postpone the increase in antimicrobial resistance. The overall use of antimicrobials must be reduced. The prudent use of antimicrobials should always be promoted. Alternative and preventive measures (such as vaccination) that can ultimately replace the use of antimicrobials should always be explored ahead of administration of antimicrobials.

## 2. ESKAPE (*Enterococcus faecium*, *Staphylococcus aureus*, *Klebsiella pneumoniae*, *Acinetobacter baumannii*, *Pseudomonas aeruginosa*, and *Enterobacter* spp.) pathogens

The acronym ESKAPE paradoxically denotes the ability of the panel of constituent bacteria, namely *Enterococcus faecium*, *Staphylococcus aureus*, *Klebsiella pneumoniae*, *Acinetobacter baumannii*, *Pseudomonas aeruginosa*, and *Enterobacter* spp., to escape the antimicrobial activities of most commonly used products in clinical treatment. The AMR capabilities of this bacterial group have been reported to severely exacerbate the condition of hospitalised patients with noncommunicable diseases, such as diabetes, cancer, cardiovascular diseases, and chronic respiratory diseases; breakage of skin barrier such as wounds; and diseases that lead to immunosuppression [4]. Exposure of these patients to ESKAPE pathogens could occur during hospitalisation through contact with medical equipment (commonly related to central line bloodstream infections, ventilator pneumonia, and urinary catheterisation), other infected patients, and healthcare staff. WHO has recently published a list of bacteria that urgently require new antibiotics and ESKAPE bacteria were identified among Priority 1 and 2 lists; as these are multidrug resistant microorganisms that pose serious threats in healthcare facilities [20].

### 2.1. Enterococcus faecium

Enterococci are gram-positive, facultative anaerobic organisms that form part of the normal intestinal flora. Among the 17 species of Enterococci, *Enterococcus faecium* and *E. faecalis* were most frequently reported to be pathogenic in humans. The pathogenic strains of these organisms cause a variety of infections, involving the endocardium, urinary tract, prostate, intra-abdominal organs, skin (particularly if present within a wound) [5]. If bacteria enter the normally sterile bloodstream through parenteral injections, catheterisation, surgery and open wounds, this can result in metastatic or systemic infections that eventually result in sepsis and septic shock. Research has shown that various virulence factors are present on the capsule, cell wall, membrane and within the cytoplasm of *Enterococcus faecium* that contributed to AMR and pathogenicity of the organism through formation of a protective and persistent biofilm, ß-lactamase production, and proteins directed against recruited inflammatory cells [6,7]. Reports submitted to the National Healthcare Safety Network (NHSN) at the Centres for Disease Control and Prevention (CDC) indicated that the *Enterococcus* group is the second most common pathogen across all hospital-acquired infections types (HAI) types. While *Enterococcus faecium* was ranked among the top ten organisms across all types of HAI, including central line-associated bloodstream infection (CLABSI), catheter-associated urinary tract infection (CAUTI), and surgical site infections (SSIs) [21].

### 2.2. Staphylococcus aureus

*Staphylococcus aureus* is a Gram-positive coccus that is frequently isolated from the skin, respiratory tract, and female lower reproductive tract as it forms part of the normal flora on the human body [8,9]. *S. aureus* infection has shown increased resistance towards penicillin, which led to the introduction of methicillin for the treatment of *S. aureus* showing resistance to penicillin in 1960 [10]. However, *S. aureus* developed resistance towards methicillin, thus giving rise to methicillin-resistant *S. aureus* (MRSA) clones [11]. MRSA is the second most common cause of HAI in the USA according to a report from the CDC [12]. The ability of the organism to form biofilms on tissues such as the skin and inert indwelling device surfaces such as intravenous catheters and surgical implants, further exposes susceptible individuals as the most common route of MRSA transmission is through direct contact [13]. Additionally, *S. aureus* can invade host cells and evade the antimicrobial effects of administered therapies [20,22]. Successful treatment of MRSA infection is restricted by worldwide antibiotic resistance towards first-line therapies such as vancomycin and teicoplanin [13]. Together, these characteristics allow this microorganism to remain an important pathogen and alternative therapeutic measures are critically needed.

### 2.3. Pseudomonas aeruginosa

*P. aeruginosa* is a Gram-negative, rod-shaped, facultative anaerobe that is ubiquitous in the environment and forms part of the normal gut flora. *P. aeruginosa* is capable of forming biofilms on medical device surfaces, thus patients dependent on breathing machines or fitted with an invasive device such as a catheter are at risk of severe and life-threatening illness [23,24,25]. Reported illnesses include endocardial valve infection through endocardial tubes, ventilator-associated pneumonia (VAP) and CAUTI. Additionally, *P. aeruginosa* has also been reported to be able to grow in intravenous fluid and could enter the bloodstream and cause sepsis [26,27,28]. The emergence of extremely drug-resistant (XDR) *P. aeruginosa* towards multiple antibiotics, e.g., cephalosporins and carbapenems, increases the problem of treatment globally and clinicians need to resort to the last available medication, colistin, a polymyxin antibiotic which was avoided for the past thirty years as it has been implicated in both neuro- and nephrotoxicity [29,30,31,32].

### 2.4. Klebsiella pneumoniae

*K. pneumoniae* is a non-fastidious, Gram-negative bacillus and commonly encapsulated. Although *K. pneumoniae* is among the population of normal flora found in the mouth, skin, and intestine, it has been reported to cause infections in the lungs, urinary tract, and bloodstreams of patients from hospitals, nursing homes and other healthcare facilities [30]. This bacterium is a remarkably resilient pathogen, instead of actively suppressing many components of the immune system, it successfully evades the body’s defence mechanisms and survives in the most harsh environments. This organism can survive and grow within the intravenous fluid and form biofilm on medical devices such as the urinary catheter, leading to detrimental septicaemia in patients [33]. Furthermore, the bacteria have developed resistance towards almost all available antibiotics: fluoroquinolones, third-generation cephalosporins and aminoglycosides [34]. The emergence of the carbapenem-resistant *K. pnemoniae* strains circulating across the globe has forced the administration of colistin, an old antibiotic and considered the last available. Nevertheless, resistance towards colistin was recently reported, rendering treatment of multidrug resistant *K. pneumoniae* even more difficult [35].

### 2.5. Acinetobacter baumnannii 

*Acinetobacter baumnannii* is a Gram-negative coccobacillus, of unknown natural habitat, that causes nosocomial infections in immunocompromised patients, including bacteraemia, pneumonia, meningitis, urinary tract infection, and wound infection [36]. The *Acinetobacter* species have a great capacity of extensive antimicrobial resistance resulting from its relatively impermeable outer membrane, the presence of efflux pumps, and lack of protein channels. Additionally, the bacteria produce various enzymes, such as beta-lactamases, to render multiple antibiotics ineffective; persistent adherence to surface through biofilm formation; as well as insertion of resistance genes from other bacterial species through genomic mutation [37]. Over the past three decades, *A. baumnannii* has been reported to be resistant to most known antibiotics, even in some cases towards colistin, the last resort of antibiotics in human medicine albeit with adverse side effects [38,39,40]. A combination of antibiotic treatment using colistin methansulfonate (CMS), a carbapenem, and ampicillin-sulbactam was reported to have the highest rate of success for colistin-resistant *A. baumnannii* [40]. The source of infection could originate from numerous sites and medical equipment within the healthcare facilities, including the door handle, curtains, keyboard, etc. However, the most detrimental effects involve patients infected through treatment involving the use of ventilator and venipuncture catheterisation where the mortality rates can reach 35% [41,42].

### 2.6. Enterobacter spp.

*Enterobacter* is a genus of Gram-negative bacilli from the family Enterobacteriaceae. These bacteria are facultative anaerobes that do not form spores but may be encapsulated. Two of the most clinically relevant species are *Enterobacter aerogenes*, and *Enterobacter cloacae* [43]. The worldwide emergence of colistin resistance genes (mcr-1, -2, -3, -4, -5, -6, -7, and the latest -8) in Enterobacteriaceae bacteria has detrimentally threatened global health as this polymyxin compound is considered the last resort of treatment [44]. According to the report provided by Weiner et al., *Enterobacter* spp. are among the top 10 pathogens detected in CAUTI, CLABSI, SSI and VAP, accounting for 17,235 HAI incidence recorded at NHSN [45]. In the same report, Enterobacter spp. were found to be resistant to extended-spectrum cephalosporin (ESC4, i.e., cefepime, cefotaxime, ceftazidime, ceftriaxone); carbapenems (imipenem, meropenem, doripenem); and multidrug-resistance (MDR1) at 12.8 to 38.2%, 1.9 to 6.6%, and 2.9 to 10.2% respectively [21]. On another note, unchecked antimicrobial misuse in the livestock industry may exacerbate colistin resistance in the general population as the medication has been added in feed as a growth promoter, or for treatment and prevention of infectious diseases in many countries.

## 3. The Progress in Antimicrobial Development

The development of a new antimicrobial is a lengthy and expensive process. The big pharmaceutical companies were once the major drivers in antimicrobial discoveries, are now no longer stand at the forefront of the arena [46]. Beginning in the late 1990s, due to lack of success, low financial returns and emergence of antimicrobial resistance, these companies began to withdraw antimicrobial discovery and development from their portfolio, with the final company Novartis followed suit in July 2018 [47]. Therefore, the task for novel discovery of antimicrobials is now left to university laboratories and small and medium-sized companies [46]. Only recently, German Ministry of Education and Research has showed interest to provide funds to Global Antibiotic Research and Development Partnership (GARDP) to accelerate discovery, development and delivery of affordable antibiotics to treat Gram-negative infections [48]. Understanding that the discovery of a novel compounds is a lengthy and expensive process, utilising the current knowledge and compounds would be one approach to ensure the continuous development of antimicrobials.

## 4. Antimicrobial Polymers Are the New Generation of Antimicrobials

**Figure 2:**
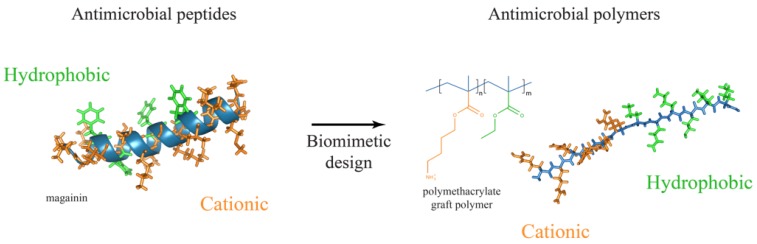
The structural similarities between antimicrobial polymers and antimicrobial peptides. Image was adapted with permission from [53].

Continuous research and understanding in the field of chemistry has opened up the possibility to design and synthesise a compound that has antibacterial activities. A new kind of antimicrobial must not only be effective against bacteria, but it must also resist the possible development of bacterial resistance. Antimicrobial polymers (AMP) are materials that have the ability to inhibit or kill the bacteria. AMP can either display the antibacterial activities through its own inherent chemical structure; e.g., quaternary nitrogen groups, halamines and poly lysine or it can serve as a backbone to improve the potency of existing antibiotics [49]. AMPs were designed based on the chemical templates provided by the antimicrobial peptides, a class of peptides of the innate immune system which protects the body from invading pathogens (Figure 2) [50]. Antimicrobial peptides (APs) are relatively small in size (10–50 amino acids), amphiphilic with cationic charge. With these physical characteristics, APs accumulate on the cell membranes and form pores on the structure, thus killing the bacteria [51]. With multimodal mechanisms of action, APs can resist acquired resistance by the bacteria. For a detailed understanding of the mechanism of action of APs, readers are invited to refer to the following article [52]. The structural and chemical diversity allow for polymer chemists to design, manipulate and construct a variety of polymers with cationic and amphiphilic structures that can function as antimicrobials. In this review, we have selected a few antimicrobial polymers that have been designed and tested against multidrug-resistant pathogens.

### 4.1. Amphiphilic Antibacterial Polymers

Advancement in natural/synthetic antimicrobial polymers exploratory has also expanded researchers’ interests in other amphiphilic polymers structures that conferred with antimicrobial activity [53]. Polymer structures and physicochemical properties such as molecular weight, polymer architecture, ratio of amphiphilic and its molecular arrangement are the potential determinants of materials’ antimicrobial potency and selectivity [54]. An ideal amphiphilic antibacterial polymer harboring a cationic arm, low molecular weight and low-level lipophilicity would likely to incur adequate antibacterial activity against Gram-positive bacteria and has minimum hemolysis activity toward human red blood cells i.e., <4% hemolysis at a given minimum inhibitory concentration (MIC) [55]. Nonetheless, Locock et al., 2014 suggested that the combinational effect offered by the specific pendant functional groups may alter the potency, selectivity and mechanisms of synthetic AMP polymers. Some empirical data showed that optimization of the degree of hydrophobicity and cationic charge is crucial for a amphiphilic polymers to attain the best antibacterial activity and minimum red blood cells haemolysis [56]. For instance, AMP-mimicking polyurethanes with a lower ratio of hydrophobic region and higher cationic strength conferred the polymers with higher bactericidal activity and lower haemolysis rate [57]. In the comparison of cationic amine- and guanidine-copolymers, the latter of low to moderate molecular weight and hydrophobicity showed higher antimicrobial activity against *S. epidermis* and lower toxicity toward red blood cells [55]. On the other hand, auto-degradation or biodegradation of polymers is an essential consideration in choosing the right antimicrobial materials [54,58]. Degradable properties of antimicrobial material avoid or minimize undesired complication caused by prolonged retention of the materials in human body or towards the environment. Controlled degradation rate of the polymers by tuning of monomer composition and amine functionality could enhance precise control of the lifespan of antimicrobial activity [47,59]. Table 2 presents a variety of amphiphilic polymers synthesized by a random synthesis approach was tested against ESKAPE pathogens. 

### 4.2. Polymers Containing Natural Peptides

**Figure 3:**
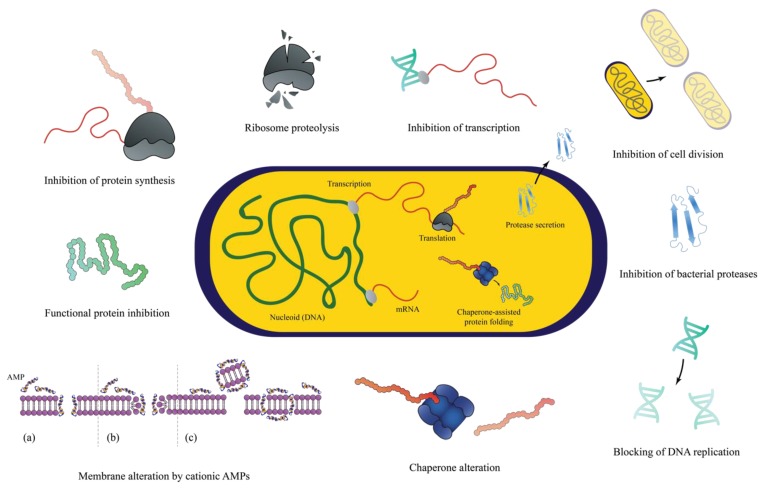
The proposed mechanism of bacterial killing activities by antimicrobial peptides. Image was adapted with permission from [69].

APs are produced by living hosts as part of innate immune responses. These protective biologics are relatively rich at various sites such as epithelial layers, phagocytic cells and body fluids such as tears and sweat [65,66]. For instance, the major epidermal APs are reported to include cathelicidins and defensins, which exhibit broad spectrum antimicrobial activities against bacterial, fungal and viral infections [67]. The mechanism of action is non-receptor dependent; and commonly activated by the alteration of bacterial membrane structure or enveloped-components, although the interruption of internal cellular function has gained increasing evidence as illustrated in Figure 3 [68,69]. In addition, APs confer a higher affinity towards negatively charge bacterial cell membranes compared to mammalian cell membranes increasing their selectivity [70,71]. However, the clinical implementation of APs are at the early stages of translation due to the following reasons: (a) susceptibility to degradation by host proteases (b) potential toxicity to the host due to high concentration needed for antimicrobial activity and (c) short half-life due to protein binding [72]. Thus, these limitations encourage the development of synthetic AMP mimics as an alternative approach.

Current studies on synthetic antimicrobial peptides focus on the compound’s susceptibility to pathogens and its toxicity effect towards the host. The susceptibility towards the pathogen is determined by the minimum inhibitory concentration of the compound that leads to either 50% (MIC_50_) or 90% (MIC_90_) bacterial growth, wherein the presence of activity was determined if the MIC_50_ or MIC_90_ was less than 100 µg/mL. Compound toxicity towards the host is determined by erythrocytosis activity. The degree of haemolytic activity of erythrocytes at MIC_50_ or the compound concentration leading to 50% red blood cell lysis determines the degree of the toxicity [70]. 

Selected studies of synthetic antimicrobial peptides and its mimics on ESKAPE pathogens are highlighted in Table 3. The model ESKAPE species were *S. aureus* and *P. aeruginosa*, as the selected Gram-positive and Gram-negative organisms, respectively. The studies showed bactericidal and/or bacteriostatic outcomes. Out of many reported compounds, only selected AMPs such as Brilacidin (also known as PMX-30063), an arylamide-peptide, has been tested in Phase II clinical trials. According to the fact sheet, a good prognosis in topical treatment of MRSA-infected wound and minimal side effects were reported [73]. Another AMP mimic, LTX 109, a membrane disrupting compound targeting Gram-positive and Gram-negative bacteria is currently under phase II clinical trials for the treatment of impetigo [73]. These two AMPs were indicated for topical used only, owing to the uncertainty from the aforementioned AMP limitations.

### 4.3. Halogen-Containing Polymers

**Figure 4:**
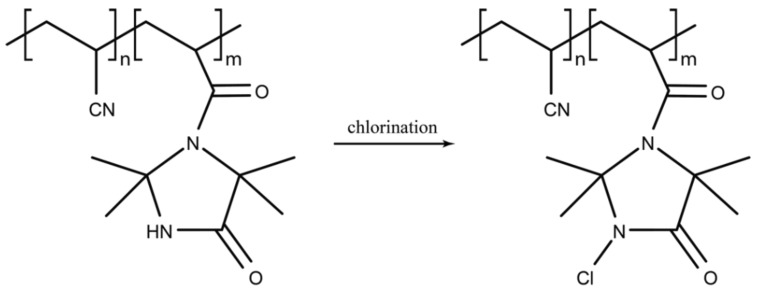
Chlorine-containing polymer.

Halogen (fluorine, chlorine, bromine, iodine) containing polymers with antimicrobial properties are attractive materials based on unique properties afforded by the associated halogen (Figure 4). Halogen-containing antimicrobials are well described. Several antimicrobials produced by *Streptomyces* spp. contain chlorine; chloramphenicol, chlortetracycline and vancomycin. Fluoroquinolones, effective against Gram-positive and Gram-negative organisms, are a class of antimicrobials where fluorine is incorporated within the quinolone structure [83]. 

Polymers containing fluorine offer antimicrobial activity due to their hydrophobic nature. A review by Munoz-Bonilla et al. highlights the use of such polymers, including the creation of a polymeric fluorine containing surfactant known as Quaterfluo^®^ [67]. The surfactant subunit alone showed robust antimicrobial activity against *P. aeruginosa*, *S. aureus*, *C. albicans* and *A. niger* [74]. When formed as a polymer, the activity against *S. aureus* increased with the length of the active perfluorakyl chains [74]. Recently, cationic fluorinated polymer emulsions have been used to create antibacterial fabrics where the presence of fluorine greatly enhanced both the antibacterial and anti-adhesion properties of the material when tested against *E. coli* and *S. aureus* [75]. 

Fluorinated polymers containing antimicrobials such as ciprofloxacin, a second-generation fluoroquinolone, have been investigated to improve solubility and bioavailability. Mesallati and colleagues prepared amorphous solid dispersions (ASDs) of ciprofloxacin with acidic polymers such as Eudragit L100, Carbopol and hydroxy propyl methyl cellulose acetate succinate (HPMCAS) [76]. When incorporated in the polymer matrix, the solubility of ciprofloxacin was improved both in water and in simulated intestinal fluid. When tested against *E. coli*, *S. aureus*, *P. aeruginosa* and *K. pneumoniae*, formulations with HPMCAS drastically improved MIC and MBC concentrations as compared to monomeric ciprofloxacin [76]. Conjugation of ciprofloxacin with polymers have also been used to create biomedical nanomaterials for use in wound dressings [77]. Ciprofloxacin-PLA (poly l-lactic acid) conjugated polymers increased the solubility of ciprofloxacin for use in the fabrication of biodegradable non-woven nanofibers by electrospinning. ciprofloxacin released from the PLA non-woven nanofibers was effective at inhibiting the growth of *E. coli* and *S. aureus* indicating the utility of ciprofloxacin conjugated polymers as an antimicrobial biomedical material [77]. 

An effective antimicrobial group of polymers contain *N*-halamines, in which at least one nitrogen-halogen covalent bond is formed by the chlorination of imide, amide and amine groups. A new *N*-halamine, hydantoin acrylamide (HA), copolymerised with siloxane (SL) to create PHASL, was used to coat cotton fabric which was shown to be effective against *E. coli* O157: H7 and *S. aureus* within 5 min once activated by chlorination [78]. More recently the halogenated 2,2,5,5-tetramethyl-1,3-imidazolidinone (TMIO) by chlorination to form 1-chloro-2,2,5,5-tetramethyl-4-imidazolidinone (MC) was found to be effective in reducing bacterial colony-forming units (CFU) of both *S. aureus* and *P. aeruginosa* when coated on wound dressings. Bactericidal activity was observed in 15 min for *S. aureus* (6-log reduction) and within 30 min for *P. aeruginosa* (7-log reduction) [79]. In this form, MC was also found to be stable when stored in the dark for 6 months, highlighting an additional utility of *N*-halamine-coated medical materials designed for wound dressings. 

### 4.4. Polymers Containing Phosphor and Sulfo Derivatives 

**Figure 5:**
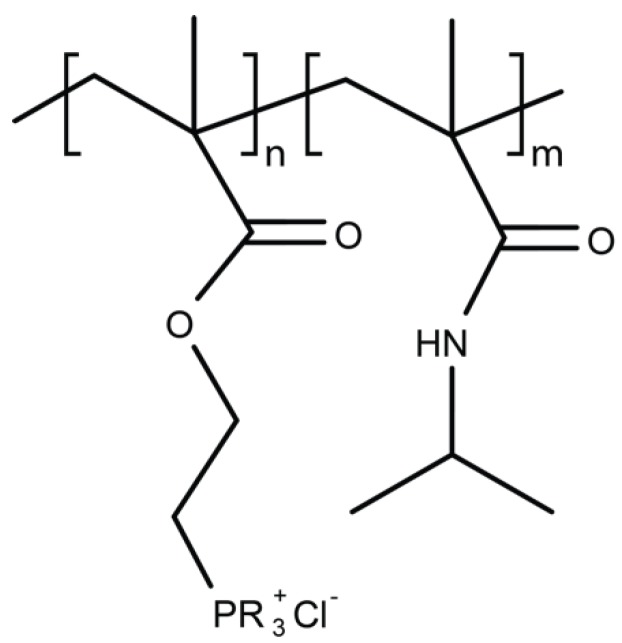
Polymer-containing phosphor-derivatives.

Polyphosphonium and polysulfonium are phospo- and sulfo- containing polymers, respectively (Figure 5). They share their mode of action with polymers comprising quaternary ammonium in causing damage to the bacterial cell wall. Phosphonium containing polycationic agents are typically considered to be more microbicidal than quaternary ammonium salt polymers with their antimicrobial potency positively related to the number of phosphonium units within the polymer [67]. Until recently, polyphosphoniums studies have been restricted to alkyl and aryl derivatives with the polymers exhibiting both hydrophobic and hydrophilic domains—a feature considered to be required for antimicrobial activity. Cuthbert and colleagues unexpectedly discovered that control polymers lacking hydrophobic alkyl chains exhibit high antimicrobial activity against *E. coli* and *S. aureus* while at the same time low lytic action on erythrocytes [80]. These findings were uncovered when investigating the ability of ‘baited’ phosphonium polymers to exert increased microbicidal effects, taking advantage of bacterial affinity to mannose sugars [80]. This work challenges the assumed requirement for balanced hydrophilic and lipophilic components within biocidal polymers and warrants further investigation into this promising class of antimicrobial polymers. 

### 4.5. Phenol and Benzoic Derivative Polymers

**Figure 6:**
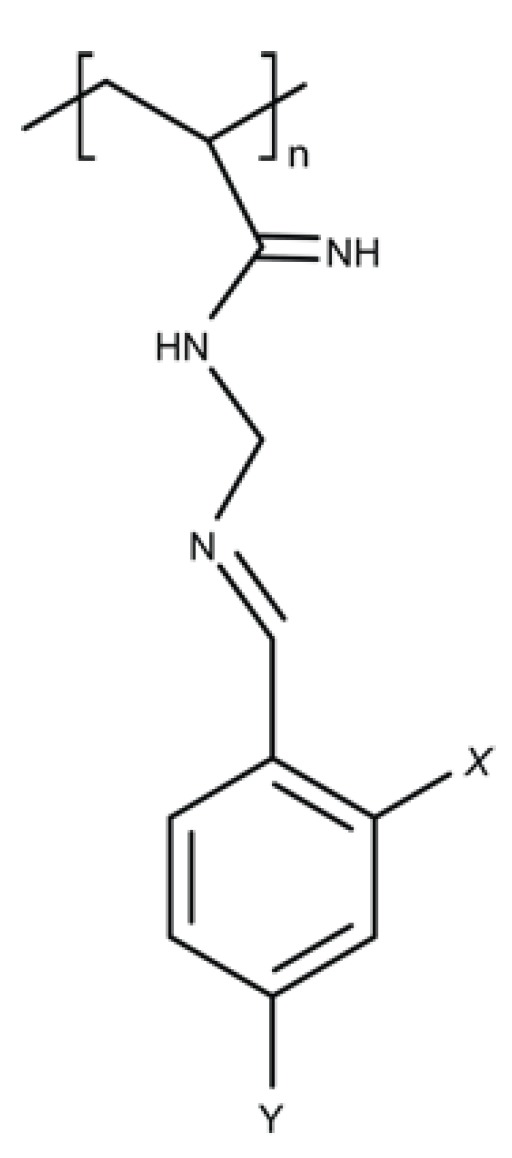
Polyacrynitrilbenzaldehyde.

Polymers containing the organic and aromatic compounds such as phenol and benzoic acid have intrinsic antimicrobial properties (Figure 6). Phenol is a strong antimicrobial agent able to disrupt the cellular membrane, while benzoic acid has broad spectrum inhibitory activity and is used as an environmentally safe antimicrobial. Vinyl polymers containing phenol or benzoic acid pendant groups were synthesised by Park and colleagues and tested for activity against *S. aureus* and *P. aeruginosa* [81]. While the polymers exhibited lower antimicrobial activity than the monomer when tested by halo diffusion, they have been used as coating materials. Nevertheless, phenol pendant vinyl polymers were marginally more effective against *S. aureus* than *P. aeruginosa* while the converse was observed for benzoic acid pendant vinyl polymers [81]. In contrast, aminated polyacrylonitrile (PAN) polymers where benzaldehyde derivatives were immobilized via their amine-terminal, were found to exhibit increased antimicrobial activity with additional bioactive groups [82]. Inhibition zone diameters significantly increased with the number of bioactive groups in each prepared polymer for several microbes, including *E. coli*, *P aeruginosa*, *S. aureus* and *A. niger* [81]. 

### 4.6. Organometallic Polymers

Organometallic polymers contain metals bonded to at least one organic molecule carbon by pi-bonds, by coordination bonds or by sigma-/pi-bonds to other elements. Antimicrobial formulations of organometallic polymers are extensive and are reviewed in depth in [67]. Silver-containing polymers are prevalent, having potent antimicrobial activity in solid form across a wide range of organisms including key ESKAPE organisms; *E. coli*, *S. aureus*, *P. aeruginosa* and *A. niger*. The same polymers in aqueous solutions, however, showed reduced efficacy in Gram-positive bacteria and yeasts [67]. Recently, Awad and colleagues took a novel approach by creating Eco-friendly silver-polystyrene nanocomposite using touline extracted orange peel to reduce silver nitrate to silver nanoparticles (AgNP) before creating a polymer with polystyrene. Antibacterial activity was observed by disk diffusion against *E. coli*, *K. pneumoniae* and *S. aureus* for both AgNP and AgNP/polystyrene polymers, although reduced for the latter in comparison [83]. Creating AgNP via this simple method, requires further investigation in the quest for novel, environmentally sound biomaterials. In general, those polymeric resins containing Cu(II), in comparison to other metal ions, show enhanced antimicrobial activity against a range of microorganisms which has been attributed to the stability of the Cu(II) ion [67]. 

### 4.7. Metal Nanoparticles Included in Polymeric Carriers 

#### 4.7.1. Polymeric Systems Containing Silver Nanoparticles

Silver nanoparticles (AgNPs) alone have a well-established use in the treatment of bacterial infections. AgNPs show an efficient antimicrobial property due to their extremely large surface area, which provides better contact with microorganisms [84]. Wen-Ru et al. have studied the antibacterial activity and acting mechanism of AgNPs on *E. coli* [85,86]. The authors investigated the growth, permeability, and morphology of the bacterial cell wall following the treatment with AgNPs. Their results showed that, based on transmission electron microscopy (TEM) imaging, the bacteria membrane vesicles were dissolved and dispersed, and the membrane components became disorganized and scattered from their original ordered and close arrangement [87]. These observations suggested that AgNPs may damage the structure of the bacterial cell membrane and depress the activity of some membranous enzymes. Studies have also shown that AgNPs are able to interact with sulfur-containing proteins present in the bacterial membrane, in the same way as they can interact with phosphorus groups present in the cell DNA [88]. AgNPs seem to preferably attack the respiratory chain, and cell division. 

The use of AgNPs in biomedical applications has been extended by the incorporation of AgNPs into polymeric systems forming multilayer films, polymeric nanotubes and nanofibres, and polymeric gels. The development of organic−inorganic hybrid nanomaterials has allowed the combination of the tunable properties of soft nanomaterials with the unique optical and electronic properties of metal nanoparticles. Due to their tunable surface, morphology and porosity, soft organic materials such as polymers that are derived from various synthetic or natural compounds can easily incorporate metal nanoparticles of different shapes and sizes. A large number of polymers have been investigated for this purpose. AgNPs can be synthesized in situ using the polymer matrix as a reaction medium, or alternatively, the AgNPs are prepared ex situ and then incorporated into the polymeric matrix [89,90,91].

Numerous studies have been carried out using different polymer systems [91,92,93,94]. Sanchez-Valdes et al. have prepared multilayer films of polyethylene and AgNPs and evaluated their antimicrobial activity towards *Pseudomonas oleovorans* and *Aspergillus niger* [95]. The authors showed that the release of the Ag^+^ ions, and therefore the efficacy of this system, was dependent on the size of the AgNPs. Other authors have focused their attention on the modification of the nanocomposite surface, at the nanometer level, by combining the effects of oxygen plasma treatment and silver nanoparticles on the poly(lactic-co-glycolic acid) (PLGA) polymer matrix [96]. In this study, PLGA nanocomposite films were produced by solvent casting with 1 wt% and 7 wt% of AgNPs. The PLGA (used as a control) and PLGA/Ag nanocomposite surfaces were then treated with oxygen plasma. Antibacterial tests were performed using *E. coli* and *S. aureus*. The plasma-treated PLGA/Ag^+^ system showed the best bactericidal effect in comparison to untreated PLGA/Ag^+^ or oxygen plasma-treated PLGA matrix for both strains [96]. 

A different approach to prepare antibacterial coatings has been described by Taheri et al. where AgNPs were encapsulated into a phospholipid bilayer and their surface immobilised to a functional plasma polymer for application on medical devices such as catheters and wound dressings. The antibacterial efficacy of the coatings was evaluated against *S. aureus*, *S.epidermidis* and *P. aeruginosa*. The innate immune response was studied in culture of primary bone marrow-derived macrophages (BMDM) and the potential cytotoxicity was assessed in culture of primary human fibroblasts. The authors also observed a reduced expression of pro-inflammatory cytokines from BMDM which suggested a reduced inflammatory response. The prepared coatings were able to reduce the growth of *S.aureus* and *P. aeruginosa* by 70% and 80%, respectively, while colonization by *S. epidermidis* was almost completely inhibited. 

**Figure 7:**
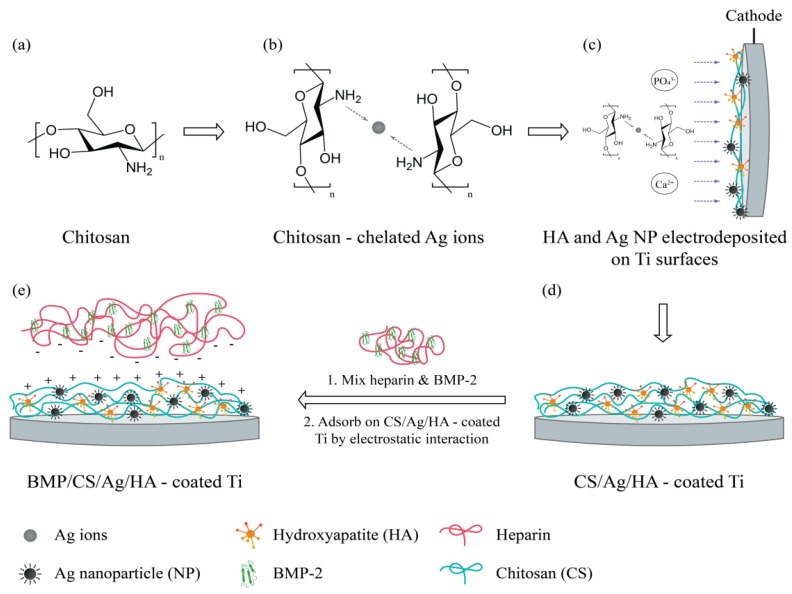
Schematic representation of the electrochemical deposition process and immobilization of bone morphology protein-2 (BMP-2_ on HA coatings on a Ti metal surface). Image was adapted with permission from [97].

Studies have also moved towards the inclusion of additional materials to the AgNPs-polymer containing systems. An example is the incorporation of growth factors (bone morphology protein-2, BMP-2) and AgNPs into hydroxyapatite (HA) coatings on metallic implant surfaces for enhancing osteo-inductivity and antibacterial properties. In this complex system, BMP-2 and AgNPs containing HA coating were prepared on titanium (Ti) surfaces by electrochemical deposition (ED). In addition, chitosan (CS) was used as a stabilising agent for the generation of the AgNPs, and simultaneously reduced their toxicity. A schematic representation of this system is shown in Figure 7. Results of antibacterial tests indicated that the CS/Ag/HA coatings have high antibacterial properties against both *S. epidermidis* and *E. coli*. Additionally, bone marrow stromal cells (BMSCs) culture results indicated that the BMP/CS/Ag/HA coatings have good osteoinductivity and promote the differentiation of BMSCs. Implantation of Ti bars with BMP/CS/Ag/HA coatings into the femur of rabbits showed that BMP/CS/Ag/HA coatings favour bone formation in vivo. Other studies can be found in the literature reporting the inclusion of other biological or synthetic compounds into the AgNPs-polymers systems such as bovine serum albumin and tiopronin.

#### 4.7.2. Nanofiber Systems Containing Silver Nanoparticles 

The development of hybrid organic–metallic systems containing AgNPs embedded in nanofibers has gained increasing interest due to the dual benefits of each individual system. Due to their high surface-to-volume ratio, polymeric nanofibers can provide a larger number of reaction sites and higher permeability. By embedding AgNPs into polymer nanofiber matrices, the composites are promising candidates for scaffolding biomaterials with antimicrobial properties [85]. 

Nanofibers are mostly prepared by electrospinning. Either in combination with other polymers, or on its own, biodegradable polymers such as PLGA, poly-caprolactone (PCL) and chitosan can be electrospun and further functionalised to achieve the desired antibacterial properties [86]. Other studies reported the one-step fabrication of silver nanoparticles embedded into poly(2-(tert-butylaminoethyl) methacrylate) (PTBAM) nanofibers by radical-mediated dispersion polymerization. PTBAM is a cationic polymer, which may increase the antimicrobial properties of the nanofibers loaded with AgNPs against Gram-negative *E. coli* and Gram-positive *S. aureus* [98]. 

A few interesting studies have used bacterial cellulose functionalized with AgNPs for wound-healing treatment [98,99]. Bacterial-derived cellulose, commonly known as bacterial cellulose (BC), is produced by the fermentation of Gram-negative bacterium *Acetobacter xylinum*, which can produce high aspect ratio nanofibers, with three-dimensional (3D) porous networks Authors showed that AgNPs sized from 5 to 12 nm, with narrow size distribution, were successfully deposited on the BC matrix. The studies showed a slow release of the AgNPs from the BC matrix, with the antibacterial effect lasting after 7 days.

#### 4.7.3. Hydrogels Containing Silver Nanoparticles

Hydrogel-based dressings containing antimicrobial components have seen an increase in research activity in wound-care applications. Boonkaew B. et al. have prepared 2-acrylamido-2-methylpropane sulfonic acid sodium salt hydrogels containing AgNPs via ultraviolet radiation [100]. None of the hydrogels were found to be toxic to any of the tested cell lines. The measurement of cumulative release of silver indicated that 70–82% of silver was released within 72 h. The antibacterial activities against Gram-positive *S. aureus* showed a log reduction >3 after 6 h of treatment. In the case of Gram-negative *P. aeruginosa*, the results showed faster inhibition as the log reduction was >3 within 3 h. The fact that Gram-positive bacteria are less susceptible to silver ions than Gram-negative bacteria could be related to a) the fact that the cell wall of Gram-positive bacteria is thicker than that of Gram negative bacteria; b) silver may get trapped by the negative charge of the peptidoglycan cell wall. 

Hydrogel based wound dressing membranes have been developed using a combination of the following polymers, polyvinylpyrrolidone (PVP), polyethylene glycol (PEG), agar and carboxymethyl cellulose (CMC) [101]. Silver ions were dispersed in the polymer matrix and its reduction with formation of a hydrogel and AgNPs was performed using gamma irradiation. In vitro and in vivo results showed increased cicatrisation with the presence of large quantity of fibroblasts being detected with little formation of collagen. Other studies explore the possibility of generating photo-activated in situ poly (ethylene glycol) diacrylate (PEGDA) and PVP hydrogel composite containing AgNPs with sustained anti-fouling/anti-bacterial activities [102]. The authors showed that the in situ method is more effective than the two-step method since a) there is minimal gel softening; b) less dispersal of nanoparticles; and c) lower concentration of metallic nanoparticles is needed, which reduces toxicity to cells. The ability of the AgNPs-hydrogel composite to control bacterial growth was evaluated by measuring bacterial growth rates in media immersed with the composite. The AgNP-PEGDA-PVP gel composite limited the bacterial growth even at the silver concentration of 0.2 mM. At a concentration of 10 mM, the system was able to inhibit bacterial growth over 5 days. 

#### 4.7.4. Inclusion of Other Metal Nanoparticles 

Other metal particles are also known for their antimicrobial activity, although they are relatively less studied than silver. Gold NPs (AuNPs) have exploited their unique chemical and physical properties for transporting and unloading pharmaceutical compounds. The gold core is essentially inert and non-toxic when compared to AgNPs [103]. Furthermore, AuNPs are chemically stable and allow easy surface functionalization [104]. One of the most attractive modifications is the coating of AuNPs with biocompatible polymers such as PEG and chitosan, which by creating composites with a modulate mechanical strength can improve their features as biomedical scaffolds [105,106]. 

Chitosan-AuNPs nanocomposites prepared by a solvent evaporation method showed high antibacterial activity and simultaneously low cytotoxicity. It has been shown that the molecular weight (Mw) and deacetylation degree of chitosan influences the size of the AuNPs formed. The resulting nanocomposites demonstrated total bactericidal effect against two biofilm forming antimicrobial resistant strains (*S. aureus* and *P. aeruginosa*) [107]. Other studies showed that chitosan- AuNPs systems have concentration-dependent bactericidal ability without damaging human macrophages in an in vitro infection model, causing bacterial wall damage as the killing mechanism [108]. 

Hybrid PEG-AuNPs nanocomplexes can be effectively bio-conjugated with the enzymes or proteins [109,110]. More recently, a study has demonstrated that engineered hybrid PEG-AuNPs covalently conjugated with a peptide called innate defense regulator (IDR)-1018 showed both bactericidal and antibiofilm properties at micromolar concentration bacteria [111]. The surface of the AuNPs can also be modified with molecules that serve as the main structural components of β-lactam antimicrobials, such as 6-aminopenicillanic acid (6-APA) [112]. The APA-modified AuNPs were electrospun with PCL/gelatin to obtain biocompatible antibacterial wound dressings. The antibacterial activity in skin wound healing by a dorsal wound model of a rat exposed to *E. coli*, MDR *E. coli*, *P. aeruginosa* and MDR *P. aeruginosa* was investigated. The results showed that wounds treated with Au-APA electrospun nanofibers had better wound-healing ability than gauze and PCL/gelatin nanofibers even against MDR bacterial wound infection. 

Copper nanoparticles (CuNPs) have been shown to have excellent antimicrobial properties. This could be due to an increased concentration of copper inside the cell which causes oxidative stress and forms hydrogen peroxide. Furthermore, excess copper causes a decrease of the membrane integrity of microorganisms, leading to the loss of vital nutritional cell elements, causing desiccation and eventually cell death [113]. Incorporation of CuNPs into medical grade polymers has also been described. CuNPs incorporated into polyurethane and silicone polymers displayed potent antibacterial activity against methicillin-resistant *S. aureus* and *E. coli* within 6 h [114].

Lu et al. added CuNPs to the mixture of anionic carboxymethyl chitosan (CMC) and alginate (Alg) polymers [115]. The authors found that the CMC/Alg/Cu scaffolds showed significantly improved capabilities of osteogenesis and killing clinical bacteria compared to CMC/Alg scaffolds fabricated by the same procedure but without adding CuNPs. Furthermore, in vivo studies demonstrated that CMC/Alg/Cu scaffolds could induce the formation of vascularized new bone tissue in 4 weeks while avoiding clinical bacterial infection even when the implantation sites were challenged with clinically relevant *S. aureus* bacteria.

#### 4.7.5. Inclusion of Titanium Dioxide and Zinc Oxide 

Titanium-based alloys as biomaterials have many advantages due to their lower modulus, intensive corrosion resistance and superior biocompatibility [116]. Furthermore, titanium dioxide (TiO_2_) is a chemically stable and inert material, and when illuminated, can continuously exert antimicrobial effects by the generation of superoxide and hydroxyl radicals [117]. Similarly to what has been reported for other metal NPs, TiO_2_ nanoparticles (TiO_2_NPs) have also been incorporated into polymeric systems by different techniques and their antibacterial properties demonstrated against a variety of microorganisms. Chitosan, PLA and PLGA remain the most widely used polymers for this purpose.

Fonseca et al. prepared PLA composites containing TiO_2_NPs, with a diameter of 10 nm and homogeneously dispersed in the polymer matrix. The PLA nanocomposites containing 8 wt% of TiO_2_NPs when irradiated with light and ultraviolet-A (UVA), showed a reduction of ~94.3% and 99.9% against *E. coli* and *Aspergillus fumigatus*, respectively. Toniatto, T.V. and his co-authors also used PLA polymer but in the fiber shape. They prepared electrospun PLA fibers with high loadings of TiO_2_NPs (1–5 wt%), which possessed bactericidal activity against *S. aureus*, however which showed no in vitro cytotoxicity using a L929 cell line [118]. Furthermore, studies have also evaluated the feasibility of PLGA-TiO_2_NPs composite biofilms under UV light irradiation, on wound healing in vitro, human keratinocytes (HaCaTs), fibroblasts (L929s), and bovine carotid artery endothelial cells (BECs) [87]. These results showed that the biofilms for artificial dressing applications, containing 10% TiO_2_NPs, were effective against *E. coli* and *S. aureus* and had a good biocompatibility on HaCaTs and L929s, however they had some cytotoxic effects on BECs. 

An interesting study of porous scaffolds of collagen and chitosan-containing TiO_2_NPs were evaluated as tissue engineering for wound repair. The collagen–chitosan composite scaffolds with various concentrations of TiO_2_NPs were prepared by freeze-drying technique. The scaffolds showed an inhibitory effect on *S. aureus*, good permeability and it may provide a humid environment for wound repairing [119]. Chitosan has been used in other studies combined with TiO_2_NPs for antibacterial applications [120]. Other binary polymer combinations containing poly(ether ether ketone) (PEEK)/poly(ether imide) (PEI) blends reinforced with bioactive TiO_2_NPs were fabricated via ultrasonication followed by melt-blending [121]. The nanocomposites showed significant antibacterial properties against human pathogenic bacteria with and without UV illumination, and the effect on *S. aureus* was systematically stronger than that on *E. coli*. 

Zinc oxide (ZnO) nanoparticles (ZnONPs) have been widely investigated thanks to its multifunctional properties coupled with the ease of preparing various morphologies, such as nanowires, nanorods, and nanoparticles [122]. Three-dimensional and interconnected porous granules of nanostructured hydroxyapatite (nanoHA) incorporated with different amounts of ZnONPs have been prepared by a simple polymer sponge replication method [123]. Granules loaded with 2% ZnONPs showed a strong antibacterial effect against *S. aureus* and *S. epidermidis*. In vivo studies used nanoHA porous granules with and without ZnONPs implanted into the subcutaneous tissue in rats and their inflammatory response after 3, 7 and 30 days was examined. The results showed the potential of these systems in reducing bacterial activity in vitro and in vivo, with a low cell growth inhibition in vitro and no differences in the connective tissue growth and inflammatory response in vivo. Sustained release preparations of polymers containing ZnONPs have been described by the formation of crosslinked polymer networks with ZnNPs in the form of hydrogels [124]. 

### 4.8. Dendrimers

**Figure 8:**
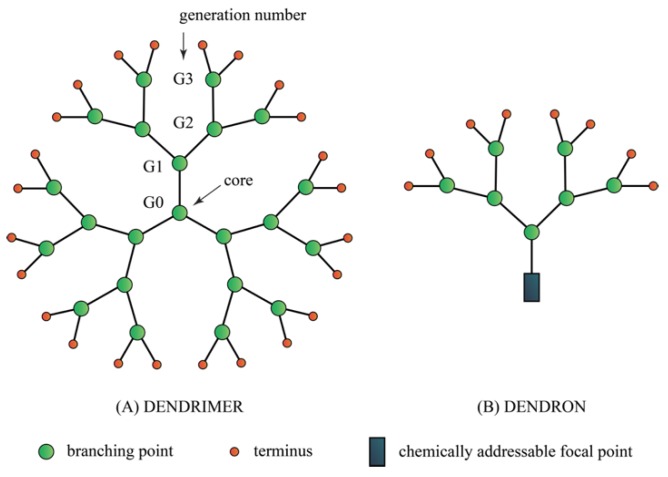
The physical structure of a dendrimer.

**Figure 9:**
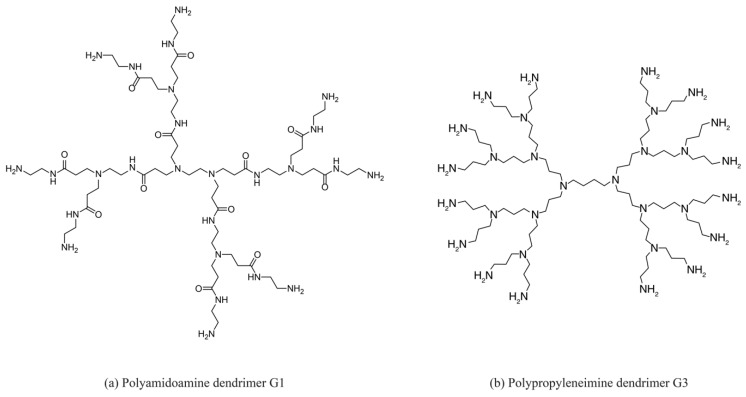
Examples of common dendrimers for biological application.

Dendrimers are a class of molecule and the word is derived from dendron which means tree for their characteristic branch-like appearances (Figure 8). The most common dendrimers used for biological applications are based on polyamidoamines (PAMAM) and polypropylene imine (PPI), (Figure 9). Dendrimers are synthesized from the core and develop into a globular structure with the size between 2–5 nm. The core structure provides attachment of the dendrons and each section represents a generation (G1, G2, G3) (Figure 8). The higher generation the dendrimer is built of, the more branched and exposed number of end groups are available for conjugation with other molecules including small molecule antibiotics [125]. Dendrimers’ chemical branches can be tailored according to the solubility and degradability to enhance biological activity of interest [126]. 

Dendrimers can demonstrate its own antibacterial activities through interaction with the bacterial lipid bilayer and causes destablisation of the bacterial structure [127]. Mofrad et al., 2018 demonstrated the efficacy of G3-poly-amidoamine dendrimer (G3-PAD) against seven species of Gram-negative and -positive bacteria. Interestingly, the highest sensitivity was observed for *Salmonella* species with the least susceptibility were observed in *Klebsiella* species. The author suggested differences in the bacterial membrane composition contributed to the strong barriers against the entrance of dendrimers inside the bacteria [128]. Additionally, Pires et al. 2015 demonstrated effective antimicrobial activities of peptide dendrimer, G3KL against multidrug resistant *Acinetobactor baumanii* and *P. aeruginosa* [129]. These two examples are only selected research on dendrimers’ antibacterial activities and readers are invited to refer more on related information as provided by [125]. Due to the multi- functional group within dendrimers, the molecules can also be conjugated with existing antibiotics to potentiate their activities. Interestingly, stimulus-controlled antibiotic released from the dendrimer can be achieved by specific triggers, for example, light, pH or temperature. Some examples of the successful functionalization of antibiotics are summarized in Table 4.

### 4.9. Polymer-Based Guanidine

**Figure 10:**
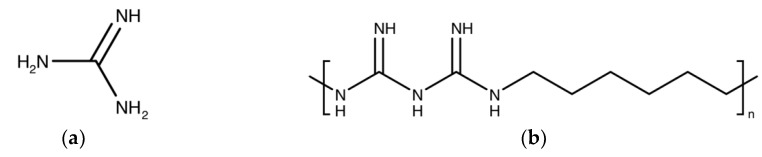
(**a**) The guanidine structure (**b**) The polyhexamethylene biguanide (PHMB) structure. PHMB is a cationic polymer of repeating hexamethylene biguanide groups, with *n* average = 10–12 (*n* is the number of structural unit repeats) and molecular weight (mw) 3025 g/mol.

**Figure 11:**
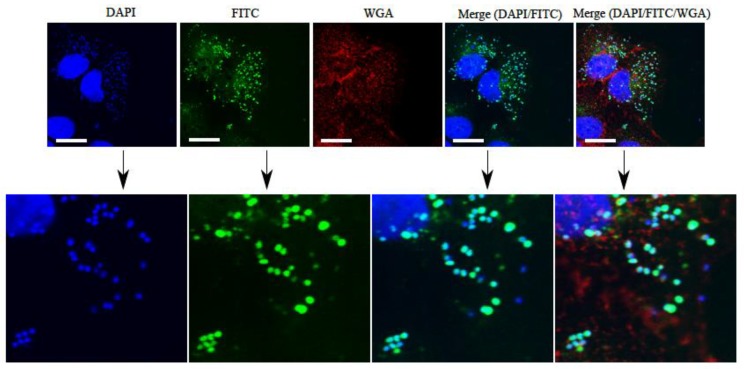
Intracellular localization and bactericidal activities of PHMB against intracellular methicillin-resistant *S. aureus* (MRSA). Colocalization of fluorescence-tagged PHMB (PHMB-FITC) with intracellular *S. aureus* strain EMRSA-15 in keratinocytes. Keratinocytes were infected with EMRSA-15 followed by treatment with PHMB-FITC (green). Keratinocytes were labelled with DAPI (blue) for keratinocytes and EMRSA-15 nuclei staining and WGA (red) for keratinocyte membrane stain. Upper panels are images of infected cells and merged images. Lower panels are enlarged images that clearly show colocalization between PHMB-FITC (green) and EMRSA-15 (blue). White scale bar is 25 μm. Image is reprinted from [22].

Guanidine-like compounds have been investigated for the past three decades and have given benefit in diverse medicinal field (Figure 10a) [138,139]. Guanidine can be found in natural terrestrial and marine environments such as microorganisms, plants and invertebrates [140]. Guanidine displays cationic properties and this allows for interaction with the anionic counterpart. The side chain diversity of guanidine allows for further development of guanidine scaffold for different therapeutic purposes [141]. Compounds containing guanidine have attracted interest and have been successfully applied as therapeutics for the central nervous system, anti-inflammatory agents, anti-thrombotic agents, anti-diabetic agents and antimicrobial agents [138]. One of the most commonly used antimicrobial polymers is polyhexamethylene biguanide (PHMB) or also known as polyhexanide (Figure 11). PHMB is synthesized by oligomirazation of guanidine salts and hexamethylenediaemine [141]. PHMB has been widely used in domestic applications, as an antiseptic in medicine, and in the food industry. PHMB applications include impregnation of wound dressings, water treatment, mouthwash and disinfection in contact lenses [142,143,144].

PHMB is a potent topical antimicrobial against Gram-positive and Gram-negative bacteria, fungi, parasites and viruses [144,145,146,147,148]. PHMB antimicrobial activities involve the interaction of biguanide groups with the cytoplasmic membrane, lipopolysaccharide and peptidoglycan of the bacterial cell wall. This binding is believed to displace the divalent cation Ca^2+^ causing membrane destabilization and cellular leakages [145]. Simultaneously, the hexamethylene segment can interact with phospholipids on the membrane, causing a phase separation that disturbs random distribution of lipids, further destabilizing the membrane structure. Furthermore, Chindera et al. demonstrated that PHMB enters bacteria cells and this leads to chromosome condensation [148]. Therefore, PHMB may have at least two mechanisms of action, and this may help to explain why acquired antimicrobial resistance to PHMB has not yet been reported, despite being used in the clinic and domestic application for the past 40 years.

PHMB has also been reported to enter mammalian cells and kill intracellular bacteria (MRSA) and parasites (*Leishmania* spp.) at low dosage (Figure 11) [22,149]. Additionally, due to the cationic nature of the molecules, PHMB is able to form nanoparticles with other small antibiotic molecules thus potentiate its antibacterial activities [20,148,149]. PHMB is considered less toxic towards mammalian cells compared to other compounds in its class. Muller and Kramer, 2008 compared the cellular cytotoxicity of PHMB and other commonly used antiseptics in the clinic and found that PHMB has a higher biocompatibility index towards mammalian cells than other antiseptics tested [150].

## 5. Challenges in Bringing Antimicrobial Polymers into Clinics

Now comes the main question as to whether bench to bedside translation of these antimicrobial polymers would come into reality or remains as the research output and ends only in the publication repository. Before choosing to embark to the clinical journey, it is mandatory for the researcher to assess the in-vitro and in-vivo toxicity, biocompatibility, evaluation of the cell viability, biodistribution, immunogenicity in order to reach the go/no go decision. Mignani et al. provide an excellent guideline on the translational requirements for dendrimers and nanoparticles to move the compounds towards the investigational new drug (IND) application, which is the evaluation of the safe profile before initiating clinical trials, the first essential step to entering the clinic phase [139]. We believe the guideline is also applicable for antimicrobial polymers and readers are invited to refer to this excellent review for comprehensive information [139].

Once the researcher establish the risk/benefit ratio, it is a wise decision to understand from the early stages the potential route of application for the compound. If the compound shows good efficacy and has potential for development as oral drugs, the researcher must understand what are the suitable requirements for the oral route, and understand the Lipinsky rule of five could be a good start [151]. However, based on the history of antibiotics development and commercialisation, antibiotics generally do not obey these rules [152]. If the compound turns out to be less likely to be developed in the oral route, other routes of administration can be an alternative, for example, topical. The guideline for development of a topical product are extensively reviewed by Chang et al. 2015 [153].

## 6. Conclusions and Future Considerations

We are living in an era where the globe is increasingly connected with highly mobile populations, moving easily between different countries, which is a compounding factor in the global AMR challenge [154]. Indeed, in 2016 at a UN General Assembly, countries worldwide affirmed their commitment to develop national action plans based on the WHO Global Action Plan for Antibiotic Resistance (2015) [2]. In principle, the plan aims to ensure “treatment and prevention of infectious diseases with quality-assured, safe and effective medicines”. A key relevant objective within the proposed WHO action plan is to strengthen knowledge through surveillance and research.

In order to provide a tangible new medicine as part of the solution for the increasing AMR problem, we propose that a network based multidisciplinary approach is vital. A continuous collaboration between chemists, microbiologists and clinicians is paramount to further develop and translate promising antimicrobial polymers for use in the clinic. One such polymer group to highlight is those containing guanidine; specifically, polyhexamethylene biguanide (PHMB) and its nanoparticle constructs, and this has been used for topical/local application. Despite PHMB being in use both for clinical and domestic purposes for over 40 years, there are no reports of resistance to date. Lack of acquired resistance is likely due to multimodal antimicrobial [145,148]. PHMB is particularly interesting for exhibiting broad spectrum activity against intracellular bacteria and parasites in nanoparticle form. Of note, PHMB demonstrates antimicrobial effects at low concentrations and has been shown to have lower toxicity than other agents of its class [20,150,155]. Future studies exploring the delivery options for PHMB, novel formulations with small molecule antibiotics, and interactions with ESKAPE isolates are required.

Clearly, going forward, the success of any existing, novel or reformulated antimicrobial of the polymer class must (i) be effective at controlling infection, (ii) exhibit low toxicity towards the host and (iii) ideally harness a multimodal arsenal of antimicrobial activity in order to provide long-term sustainability. Finally, we call on national research funding agencies to continue their commitment to the global AMR challenge. Strategic, allocated funding will play a key role in supporting the development and in vivo application of novel polymeric systems to treat resistant microbial infections that, currently, available therapeutics are failing to treat.

## Figures and Tables

**Table 1:** The global prevalence of AMR among ESKAPE (*Enterococcus faecium*, *Staphylococcus aureus*, *Klebsiella pneumoniae*, *Acinetobacter baumannii*, *Pseudomonas aeruginosa*, and *Enterobacter* spp.) pathogens.

	Pathogens	*Enterococcus faecium*	*Staphylococcus aureus*	*Klebsiella pneumoniae*	*Acinetobacter baumannii*	*Pseudomonas aeruginosa*	*Enterobacter* spp.	References
Country								
Thailand		-	-	ER	ER	-	-	[4]
South India		-	-	-	-	S	-	[5]
India		ER	ER	ER	ER	ER	ER	[6]
India (Veterinary Cases)		ER	ER	ER	ER	ER	ER	[7]
Iran		ER	HR	R	ER	ER	HR	[14]
Asia-Pacific		-	-	R	ER	S	S	[8]
Southern Italy		S	R	S	ER	R	-	[9]
Romania		HR	R	HR	HR	HR	-	[10]
Romania		H	ER	ER	ER	S	-	[11]
South Africa		R	S	ER	H	S	-	[12]
Brazil		ER	ER	HR	HR	ER	ER	[13]
Latin-America		-	-	R	ER	S	S	[8]

**-** (N/A) = data on the AMR prevalence are not available in the study, S (Susceptible) = 50% of the organism does not show resistance against any antibacterial agent; R (Resistant) = 50% of the organism must show resistance against an antibacterial agent from 1 of the antibiotic groups; HR (Highly Resistant) = 50% of the organism must show resistance against antibacterial agents from at least 2 of the antibiotic groups; ER (Extremely Resistant) = 50% of the organism must show resistance against antibacterial agents from at least 3 of the antibiotic groups. Antibiotic groups: Aminoglycosides, Carnapenems, Cephalosporins, Glycopeptides, Lincosamides, Lipopeptide, Macrolides, Monobactams, Nitrofurans, Penicillin, Fluoroquinolones, Sulfonamides, Tetracycline.

**Table 2:** Amphiphilic antimicrobial polymers activities against ESKAPE pathogens.

Polymers	Class	Description	Susceptibility						Haemolytic Activity	References
			E	S	K	A	P	E		
4-aminobutylene side chain coupled with hydrophobic ethylmethacrylate in a roughly 70/30 ratio	Amphiphilic Methacrylate Copolymers	Cationic amphiphilic random copolymers with ethyl methacrylate (EMA) comonomer were prepared with a range of comonomer fractions, and the library of copolymers was screened for antimicrobial and hemolytic activities.	BC	BS	-	BS	BS	BS	Low	[60]
PDMAEMA-g-rosin	Cationic polymers	Quaternary ammonium-containing poly(*N*,*N*-dimethylaminoethyl methacrylate) with natural rosin as the pendant group.	BS	BS	-	-	-	-	NA	[61]
Methacrylate Copolymer (E429)	Methacrylate Copolymer	Amphiphilic random copolymers with modulated cationic side chain spacer arms structure which include 2-aminoethylene, 4-aminobutylene, and 6-aminohexylene groups.	BS	BS	-	BS	BS	BS	NA	[62]
PAPMA	Amphiphilic Methacrylamide Copolymers	A series of copolymers containing lysine mimicking aminopropyl methacrylamide (APMA) and arginine mimicking guanadinopropyl methacrylamide (GPMA).	BS	BS	-	-	BS	-	NA	[63]
Cationic polyester-based copolymer	Self-Degradable Antibacterial Polymers	Auto-degradable antimicrobial copolymers bearing cationic side chains and main-chain ester linkages synthesized using the simultaneous chain- and step-growth radical polymerization of t-butyl acrylate and 3-butenyl 2-chloropropionate, followed by the transformation of t-butyl groups into primary ammonium salts.	BS	-	-	-	-	-	Low-Moderate	[58]
AMP-mimicking polyurethanes	Peptidomimetic Polyurethanes	Peptidomimetic polyurethanes with pendant functional groups that mimic lysine and valine amino acid residues	BC	-	-	-	-	-	Low	[57]
Block Amphiphilic Copolymers	Amphiphilic copolymers of Poly(Vinyl Ether)s	A series of amphiphilic block copolymers of poly(vinyl ether) derivatives prepared by base-assisting living cationic polymerization.	BS	-	-	-	-	-	Low	[64]
Random Amphiphilic Copolymers	Amphiphilic copolymers of Poly(Vinyl Ether)s	A series of amphiphilic random copolymers of poly(vinyl ether) derivatives prepared by base-assisting living cationic polymerization.	BS	-	-	-	-	-	High	[64]

Note: ESKAPE—*Enterococcus faecium*, *Staphylococcus aureus*, *Klebsiella pneumoniae*, *Acinetobacter baumannii*, *Pseudomonas aeruginosa* and *Enterobacter* spp.; antibacterial effect: BC—Bactericidal, BS—Bacteriostatic. Colour code for toxicity: White—data not available, Yellow—low haemolytic activity, Red—high haemolytic activity.

**Table 3:** Synthetic antimicrobial polymers mimicking peptides activities against ESKAPE pathogens.

Polymers	Class	Description	Susceptibility						Haemolytic Activity	References
			E	S	K	A	P	E		
Idolidicin variants	Peptide	A 13-residue cationic antimicrobial peptide (sequence carboxy-terminal amidated ILPWKWPWWPWRR-NH_2_)		BC					High	[66,73]
Gratisin analogues	Peptide	cyclo(-Val^1^-Orn^2^-Leu^3^-d-Phe^4^-Pro^5^-d-Tyr^6^-)_2_		BS			BS		Low	[69]
LL-37	Peptide	A cathelin-associated antimicrobial peptide		BS			BS		None	[74]
α/β-Peptides	Peptide	Helix-forming α/β-peptides, i.e., oligomers containing a 1:1 pattern of α- and β- amino acid residues in the backbone	BS	BS					None	[75]
cecropin/melittin	Peptide	Hybrid peptide produced by recombinant DNA technology in *S. aureus*					BC		NA	[76]
Maleic anhydride copolymers	Peptide mimics	Peptides Mimicking Copolymers of Maleic Anhydride and 4-Methyl-1-pentene		None			None		High	[77]
Brilacidin	Peptide mimics	also known as PMX-30063, a defensin-mimetic and a membrane-targeting arylamide oligomer		BC					NA	[71]
cecropin/melittin	Synthetic peptide	Recombinant hybrid peptide					BC		NA	[76]
LL-37LTX 109	Peptide mimics	a synthetic antimicrobial peptidomimetic containing a modified tryptophan derivate as lipophilic bulk, displayed a combination of high antibacterial activity against MRSA and *Staphylococcus* spp. biofilm		BC			BS		Low	[78]
poly(*m*-phenylene ethynylene)s	Peptide mimics	Nonhemolytic abiogenic polymers	BS	BS	BS	BS	BS	BS	Low	[79]
Pandinin 2	Peptide Variants	A scorpion venom AMP contains a central proline residue		BC					High	[80]
Pyridinium Functionalized Polynorbornenes	Synthetic peptide	Amphiphilic polyoxanorbornene with different quaternary alkyl pyridinium side-chains		BS					NA	[81]
Amino-Functionalized Poly(norbornene)	Synthetic peptide	Homopolymers of the amine bearing monomers and random copolymers of amine- and alkyl-substituted monomers of high average molar mass was produced by ring-opening metathesis polymerization.		BS			BS		None	[82]

Note: ESKAPE—*Enterococcus faecium*, *Staphylococcus aureus*, *Klebsiella pneumoniae*, *Acinetobacter baumannii*, *Pseudomonas aeruginosa and Enterobacter spp.*; antibacterial effect: BC—Bactericidal, BS—Bacteriostatic. Colour code for toxicity: White—data not available, Green—No haemolytic activity detected, Yellow—low haemolytic activity, Red—High haemolytic activity.

**Table 4:** Examples of dendrimers conjugated with antibiotics.

Dendrimers	Antibiotics Conjugates	Pathogens Tested	Mechanism of Antibiotic Release	References
Polyamidoamines (PAMAM)	Ciprofloxacin	*E. coli*	Light-active release	[130]
PAMAM	Vancomycin	*S. aureus*	Temperature-active release	[131]
PAMAM	Vancomycin	*S. aureus*	NA	[132]
PAMAM	Erythromycin	*S. aureus*	Hydrolysis of the ester linkage	[133]
Polypropylene imine (PPI)-modified maltose	Amoxicillin	*E. coli* and *P. aeruginosa*	NA	[134]
PPI	Ceftazidime	*P. aeruginosa*	pH-active release	[135]
Polyesters	Fusidic acid	*S. aureus*	Water-active release	[136]
Carbohydrate-glycopeptide	Tobramycin	*P. aeruginosa*	Temperature-active release	[137]
